# Supplementary material for: Identification of Potential Key lncRNAs in the Context of Mouse Myeloid Differentiation by Systematic Transcriptomics Analysis
Source: Genes (Basel). 2021 Apr 23;12(5):630. doi: 10.3390/genes12050630 (PMC8146222; doi:10.3390/genes12050630)
Supplement: Supplementary file 1 [file genes-12-00630-s001.zip › genes-1136279-supplementary/Supplementary Table S1, S2, S5, S7, S8.pdf]

Identification of potential key lncRNAs in the context of mouse myeloid differentiation by systematic transcriptomics analysis

Yongqing Lan[1,2,3], Meng Li[1,2], Shuangli Mi[1,2,3*]

[1]Key Laboratory of Genomic and Precision Medicine, Beijing Institute of Genomics, Chinese Academy of Sciences, Beijing 100101, China

[2]China National Center for Bioinformation, Beijing 100101, China

[3]University of Chinese Academy of Sciences, Beijing 100049, China

[*]To whom correspondence should be addressed:

Shuangli Mi, PhD

Beijing Institute of Genomics, Chinese Academy of Sciences

NO.1 Beichen West Road, Chaoyang District, Beijing, 100101, China

Tel: +86 10 84097730

E-mail: mishl@big.ac.cn

Supplementary Table S1. Information of samples used to gene co-expression analysis

data source	cell type	#reads	mapping %	read length	layout	platform	run
GSM1532584	HSC	18651232	84.80%	50bp	se	HiSeq 2000	WT-HSC-rep1_SRR1631794
GSM1532586	HSC	21745887	86.40%	50bp	se	HiSeq 2000	WT-HSC-rep2_SRR1631795
GSM1532588	HSC	18889652	80.00%	50bp	se	HiSeq 2000	WT-HSC-rep3_SRR1631796
GSM1532589	MLLENL-HSC	12433313	89.30%	50bp	se	HiSeq 2000	MLLENL-HSC-rep1_SRR1631797
GSM1532590	MLLENL-HSC	22311944	90.10%	50bp	se	HiSeq 2000	MLLENL-HSC-rep2_SRR1631798
GSM1532591	MLLENL-HSC	25057116	87.90%	50bp	se	HiSeq 2000	MLLENL-HSC-rep3_SRR1631799
GSM1532592	preGM	47555706	92.80%	50bp	se	HiSeq 2000	WT-preGM-rep1_SRR1631800
GSM1532593	preGM	41324382	88.50%	50bp	se	HiSeq 2000	WT-preGM-rep2_SRR1631801
GSM1532594	preGM	47856422	91.40%	50bp	se	HiSeq 2000	WT-preGM-rep3_SRR1631802
GSM1532595	MLLENL-preGM	51826915	95.60%	50bp	se	HiSeq 2000	MLLENL-preGMP-rep1-SRR1631803
GSM1532596	MLLENL-preGMP	41195154	93.40%	50bp	se	HiSeq 2000	MLLENL-preGMP-rep2-SRR1631804
GSM1532597	MLLENL-preGMP	45708980	93.40%	50bp	se	HiSeq 2000	MLLENL-preGMP-rep3-SRR1631805
GSM1894174	BM-kit	29854562	97.50%	50bp	se	HiSeq 2000	BM-kit-rep1_SRR2518239
GSM1894175	BM-kit	33035618	97.90%	50bp	se	HiSeq 2000	BM-kit-rep2_SRR2518240
GSM1894182	MLLENL-BM-kit	37565590	97.40%	50bp	se	HiSeq 2000	MLLENL-rep1_SRR2518247
GSM1894183	MLLENL-BM-kit	41765799	97.40%	50bp	se	HiSeq 2000	MLLENL-rep2_SRR2518248
SAMEA3134088	CMP	46828582	89.60%	70bp	pe	HiSeq 2000	CMP-ERR674975

SAMEA3134082	GMP	57009522	92.90%	70bp	pe	HiSeq 2000	GMP-ERR674985
SAMEA3134087	Granulo	40519666	94.10%	70bp	pe	HiSeq 2000	Granulo-ERR674984
SAMEA3134085	Mono	54883794	94.70%	70bp	pe	HiSeq 2000	Mono-ERR674981
SAMEA2768234	IT-HSC	58352796	94.70%	70bp	pe	HiSeq 2000	IT-HSC-ERR599397
SAMEA2768228	LT-HSC	37314690	93.60%	70bp	pe	HiSeq 2000	LT-HSC-ERR599387
SAMEA2768231	MPP	94130516	95.80%	70bp	pe	HiSeq 2000	MPP-ERR599392
SAMEA2768230	Macrophage	74294966	81.20%	70bp	pe	HiSeq 2000	Macrophage-ERR599391
SAMEA2768235	ST-HSC	132057254	87.20%	70bp	pe	HiSeq 2000	ST-HSC-ERR599388

Supplementary Table S2. Gene numbers in each module of gene co-expression network

module	#total gene	#coding gene	#lncRNA gene
black	3390	3214	176
darkgreen	1373	985	388
turquoise	1318	1081	237
lightcyan	1289	1222	67
midnightblue	1257	836	421
saddlebrown	1059	995	64
bisque4	1044	606	438
greenyellow	702	253	449
darkgrey	623	349	274
orangered3	570	558	12
lightsteelblue1	562	543	19
cyan	551	473	78
darkmagenta	492	404	88
firebrick4	444	348	96
darkorange2	344	191	153
royalblue	273	128	145
indianred4	248	242	6
sienna3	178	164	14
mediumpurple2	156	145	11
lightcyan1	154	144	10
skyblue1	149	145	4
salmon4	109	100	9
palevioletred3	96	82	14
maroon	91	79	12
lavenderblush3	88	68	20
coral1	87	86	1
antiquewhite4	85	71	14
coral2	80	74	6
skyblue2	77	55	22

plum	64	57	7
lightsteelblue	54	53	1
lightcoral	49	49	0
grey	5	4	1

Supplementary Table S5. 50 genes including 19 TFs and 31 other coding genes

other genes		TFs	
Ensemble ID	gene name	Ensemble ID	gene name
ENSMUSG00000003812	Dnase2a	ENSMUSG00000005583	Mef2c
ENSMUSG00000006362	Cbfa2t3	ENSMUSG00000015053	Gata2
ENSMUSG00000014599	Csf1	ENSMUSG00000020160	Meis1
ENSMUSG00000017737	Mmp9	ENSMUSG00000020644	Id2
ENSMUSG00000018925	Heatr9	ENSMUSG00000021025	Nfkbia
ENSMUSG00000020120	Plek	ENSMUSG00000021356	Irf4
ENSMUSG00000024778	Fas	ENSMUSG00000022508	Bcl6
ENSMUSG00000024789	Jak2	ENSMUSG00000022528	Hes1
ENSMUSG00000025804	Ccr1	ENSMUSG00000034957	Cebpa
ENSMUSG00000027858	Tspan2	ENSMUSG00000037465	Klf10
ENSMUSG00000029287	Tgfbr3	ENSMUSG00000038227	Hoxa9
ENSMUSG00000029434	Vps33a	ENSMUSG00000038236	Hoxa7
ENSMUSG00000030427	Lilra6	ENSMUSG00000038253	Hoxa5
ENSMUSG00000030589	Rasgrp4	ENSMUSG00000041515	Irf8
ENSMUSG00000030653	Gm45837	ENSMUSG00000044220	Nkx2-3
ENSMUSG00000031906	Smpd3	ENSMUSG00000052435	Cebpe
ENSMUSG00000032496	Ltf	ENSMUSG00000052684	Jun
ENSMUSG00000032501	Trib1	ENSMUSG00000055148	Klf2
ENSMUSG00000032750	Gab3	ENSMUSG00000076431	Sox4
ENSMUSG00000034394	Lif		
ENSMUSG00000034994	Eef2		
ENSMUSG00000037992	Rara		

ENSMUSG00000038301	Snx10
ENSMUSG00000040274	Cdk6
ENSMUSG00000041324	Inhba
ENSMUSG00000053617	Sh3pxd2a
ENSMUSG00000057729	Prtn3
ENSMUSG00000058818	Pirb
ENSMUSG00000061702	Tmem91
ENSMUSG00000096010	H4f16
ENSMUSG00000110195	Pde2a

Supplementary Table S7. Genes enriched to GO terms in figure 4b

GO Term	Count	%	P-value	Genes
I-kappaB kinase/NF-kappaB signaling	5	1.42	2.24E-03	Relb, Bcl10, Malt1, Tlr8, Tifab
regulation of I-kappaB kinase/NF-kappaB signaling	4	1.14	3.00E-03	Tnf, Card6, Cpne1, Nlrp12
negative regulation of NF-kappaB transcription factor activity	6	1.70	5.91E-03	Tnfaip3, Nfkbia, Tlr9, Nod2, Nlrp12, Brms1
myeloid cell differentiation	4	1.14	1.13E-02	Jak2, Clec5a, Cebpa, Irf8
positive regulation of tyrosine phosphorylation of Stat3 protein	4	1.14	1.51E-02	Il6st, Csf1r, Jak2, Osm
positive regulation of ERK1 and ERK2 cascade	9	2.56	1.78E-02	Il6st, Csf1r, Jak2, Osm
positive regulation of gene expression	14	3.98	2.38E-02	Rarg, Relb, Nfe2l2, Tnf, Ctsh, Smad3, Ccl5, Inhba, Id1, Tlr9, Pid1, Plaur, Srpk2, Ccr5
tyrosine phosphorylation of Stat1 protein	2	0.57	3.46E-02	Jak2, Osm
activation of NF-kappaB-inducing kinase activity	3	0.85	3.48E-02	Malt1, Card10, Chil1
positive regulation of NF-kappaB transcription factor activity	6	1.70	4.25E-02	Tnf, Bcl10, Tgfbr3, Malt1, Tlr9, Nod2
regulation of toll-like receptor signaling pathway	2	0.57	6.81E-02	Birc3, Esr1
tyrosine phosphorylation of Stat5 protein	2	0.57	8.44E-02	Osm, Jak2
hematopoietic progenitor cell differentiation	5	1.42	9.28E-02	Dock7, Rest, Inhba, Pld4, Ssbp3

Supplementary Table S8. gRNAs sequences and PCR primers

name	sequnences(5'>3'')
Gapdh qPCR Forward primer	AGGTCGGTGTGAACGGATTTG
Gapdh qPCR Reverse primer	TGTAGACCATGTAGTTGAGGTCA
Gdal1 isoform 1+2 qPCR Forward primer	TCTGAACCCAACTGAAACAGG
Gdal1 isoform 1+2 qPCR Reverse primer	CGTGGGATGCTCAGGTACA
Gdal1 qPCR isoform 1 Forward primer	AGAGAGGATGGGAATGCTGG
Gdal1 qPCR isoform 1 Reverse primer	CCTCCGGCTCTCCTGTTTAT
Gdal1 qPCR isoform 2 Forward primer	CAGCAGGGGAACAGAAGAGA
Gdal1 qPCR isoform 2 Reverse primer	CTAGAACTGGAGGCCAAGCA
Gdal1 qPCR isoform 3 Forward primer	CTTGTCACCAAGGAACGGGA
Gdal1 qPCR isoform 3 Reverse primer	TCTGGGTGAACGTGTCTCTT
Cebpa qPCR Forward primer	TGGACAAGAACAGCAACGAG
Cebpa qPCR Reverse primer	TCACTGGTCAACTCCAGCAC
Cebpe qPCR Forward primer	GCTACAATCCCCTGCAGTACC
Cebpe qPCR Reverse primer	TGCCTTCTTGCCCTTGTG
Gdal1 gRNA1 forward	CACCGTGGGGTCAGCTGAGTCTATC
Gdal1 gRNA1 reverse	AAACGATAGACTCAGCTGACCCCAC
Gdal1 gRNA2 forward	CACCGATAGATATAGGAACCCTAAC
Gdal1 gRNA2 reverse	AAACGTTAGGGTTCCTATATCTATC
Gdal1 gRNA3 forward	CACCGCTCCATCACTGAGGTAAGTC
Gdal1 gRNA3 reverse	AAACGACTTACCTCAGTGATGGAGC
Gdal1 gRNA4 forward	CACCGCTCTTACTCTGGAACTCCGC

Gdal1 gRNA4 reverse	AAACGCGGAGTTCCAGAGTAAGAGC
Gdal1 gRNA5 forward	CACCGGATTCACTTTCAGGTAGGCA
Gdal1 gRNA5 reverse	AAACTGCCTACCTGAAAGTGAATCC
Gdal1 gRNA6 forward	CACCGATTAGGGGTGTCCCGTTCCT
Gdal1 gRNA6 reverse	AAACAGGAACGGGACACCCCTAATC
Cebpe gRNA forward	GCATcgtctcACACCGCTAGGGCAAATCTA<br>G<br>GACCGGTTTTAGAGCTAGAAATAGCAAG
Cebpe gRNA reverse	GCATcgtctcAAAACTCGCAAAGAGCGCCC<br>T<br>AACTTGGGAAAGAGTGGTCTCATA
